# Global epidemiology of the factor H binding protein in meningococcal serogroup B invasive isolates: a targeted literature review

**DOI:** 10.3389/fpubh.2026.1937472

**Published:** 2026-10-12

**Authors:** Raffaella Iantomasi, Lacey Lopez, Joan Forns, Sarah Rose Slate, Jamie Findlow

**Affiliations:** 1 Pfizer, Milan, Lombardy, Italy; 2 RTI Health Solutions, Durham, NC, United States; 3 RTI Health Solutions, Barcelona, Spain; 4 Boston University School of Public Health, Boston, MA, United States; 5 Global Vaccine Medical Affairs, Pfizer Ltd., Tadworth, United Kingdom

**Keywords:** epidemiology, factor H binding protein, global, invasive meningococcal disease, meningococcal serogroup B, *Neisseria meningitidis*

## Abstract

**Introduction:**

Invasive meningococcal disease (IMD) is a relatively rare but clinically serious infection caused by the bacterium *Neisseria meningitidis*, with meningococcal serogroup B (MenB) being one of the most common causes. Both of the currently available MenB vaccines contain factor H binding protein (fHbp), which has high antigenic variability; thus, it is important to monitor MenB-fHbp epidemiology. This targeted literature review aimed to assess the global prevalence and diversity of fHbp in MenB isolates over time.

**Methods:**

A search of PubMed was conducted in June 2025 to identify studies reporting on MenB-fHbp characterization, without any search limits. Data were extracted and confirmed through independent review. Nomenclature was unified using PubMLST, and results were summarized descriptively.

**Results:**

Of 1,354 identified studies, 52 were selected for inclusion, published between 2009 and 2025, representing 34 countries across Africa, Asia, Australia, Europe, North America, and South America. MenB-fHbp subfamily A variants were typically dominant in Africa, Asia, and South America, while MenB-fHbp subfamily B variants were typically dominant in Australia, Europe, and North America. Prevalent fHbp peptides in MenB isolates varied widely across countries and regions and within countries over time, including a multitude of less common peptides.

**Conclusion:**

The studies included in this review demonstrate the global diversity of fHbp in MenB isolates, highlighting the importance of continued surveillance and the need for vaccines that provide broad fHbp coverage.

## 1 Introduction

Invasive meningococcal disease (IMD)—a relatively rare but clinically serious infection caused by the bacterium *Neisseria meningitidis* (*N. meningitidis*)*—*commonly manifests as meningitis and/or septicemia and is associated with an estimated case fatality rate of up to 15%, with up to 20% of survivors experiencing debilitating lifelong sequelae (1–4). *N. meningitidis* is classified into serogroups based on their capsular polysaccharide composition, which is key to their virulence (5). A total of 12 serogroups have been identified, with meningococcal serogroup B (MenB) being one of the most common causes of IMD globally, along with serogroups A, C, W, and Y (6, 7).

Routine meningococcal vaccination is a key public health strategy for controlling IMD caused by vaccine-preventable serogroups (8, 9). Currently available pentavalent MenABCWY vaccines consist of conjugate vaccines targeting serogroups A, C, W, and Y, which enhance immune responses by linking capsular polysaccharide antigens to carrier proteins (10), and protein-based vaccines targeting serogroup B; these vaccines differ in their antigen composition and, for conjugate vaccines, in their carrier proteins (11, 12). The polysaccharide capsule has been successfully used as a vaccine antigen only for serogroups A, C, W, and Y. As a result, subcapsular proteins have been used in MenB vaccines instead (13–15). Currently, two vaccines are approved in various countries for the prevention of IMD caused by MenB: 4CMenB (Bexsero; GSK Vaccines s.r.l., Sovicille, Italy) and MenB-fHbp (bivalent rLP2086 [Trumenba]; Pfizer Inc., Philadelphia, Pennsylvania, United States) (16, 17). Both MenB vaccines include recombinant *N. meningitidis* factor H binding protein (fHbp), a key virulence factor that facilitates immune evasion by binding to human complement factor H (15, 18). By inhibiting the activation of the alternative complement pathway, fHbp increases bacterial resistance to complement-mediated killing and supports meningococcal survival in the host (18). fHbp is highly variable (15), and this diversity may contribute to immune evasion, with important implications for vaccine design and breadth of coverage.

Each fHbp peptide is assigned a unique numeric identifier based on its amino acid sequence using the PubMLST scheme (i.e., peptide numbers with no further categorization). fHbp peptides can also be grouped into either three variants [Novartis scheme: 1, 2, and 3, with unlimited subvariants in each (e.g., 1.1, 2.1)] or two subfamilies [Pfizer scheme: subfamilies A and B, with unlimited variants (e.g., A01, B01)] (19). This review uses unique PubMLST peptide identification numbers and the Pfizer scheme because subfamilies A and B represent genetically distinct groups, and antibodies raised against one subfamily generally do not provide cross-protection against the other subfamily (14, 20–22). Differences between fHbp variants are determined by their amino acid sequences (15). The three-dimensional structure of fHbp has been shown to impact protein interactions that are important in determining immune reactions (15). Lipidated fHbp antigens have been demonstrated to induce more robust immune responses than non-lipidated fHbp proteins (23). The 4CMenB vaccine contains a single non-lipidated fHbp antigen [peptide 1 (B24)] and three other antigens, while the MenB-fHbp vaccine contains two lipidated fHbp antigens [peptide 45 (A05) and peptide 55 (B01)], thus offering broader fHbp coverage (22). This difference in breadth of coverage is important when selecting vaccines. For example, during a 2023 MenB outbreak in a nursery in England, tests indicated that the causative strain was unlikely to be covered by the 4CMenB vaccine, resulting in the decision to provide MenB-fHbp vaccination to staff and children (24). Similarly, in a 2021/2022 MenB outbreak at a university in England, the coverage of the 4CMenB vaccine was “unpredictable” against the fHbp peptide 13 (B09), which was present in 15 of the 16 outbreak isolates. However, immunogenicity data suggested that MenB-fHbp–containing vaccines would provide protection (25).

As of January 2026, more than 1,500 fHbp peptides have been recorded in the PubMLST database (26), demonstrating the diversity of fHbp; however, the database does not necessarily reflect the true frequency or epidemiology of fHbp variants, as it is dependent on the submission of sequences for curation (i.e., not all countries submit data, and many only submit data from selected isolates). A PubMLST database analysis of isolates from 2021 and earlier showed that over half of the listed MenB strains harbored one of only five prevalent fHbp variants [peptides 4 (B16), 1 (B24), 13 (B09), 19 (A22), and 14 (B03)] (22). Given its antigenic variability, it is important to continuously monitor the global epidemiology of fHbp to understand vaccine coverage and guide IMD prevention strategies.

This study aimed to determine the global prevalence and diversity of fHbp in MenB isolates over time by conducting a targeted literature review of published data.

## 2 Materials and methods

### 2.1 Targeted search strategy

PubMed was searched in June 2025 using terms for MenB and fHbp (Supplementary Table S1). No search limits were applied. Article titles and abstracts were screened for relevance, followed by full-text examination. Studies that reported MenB-fHbp characterization were included.

### 2.2 Data extraction and synthesis

Key data were extracted manually using a standardized template in Excel (Microsoft Corp.) and were confirmed through an independent review.

For each study, we extracted the total number of isolates; the proportion of fHbp subfamilies A, B, hybrid (i.e., a variant that does not fall clearly into either subfamily and includes sequence characteristics of both), or “other”; and the unique peptide variants that were reported. Nomenclature was standardized using PubMLST (26).

Descriptive results are presented, highlighting trends across countries and over time. The fHbp peptide data are reported as the PubMLST peptide number followed by the Pfizer variant in parentheses. Peptides without a Pfizer variant designation are indicated by the Pfizer subfamily followed by an asterisk (e.g., “A*” and “B*”) within the text. fHbp peptides were classified as “prevalent” if they are expressed by ≥ 5 isolates within the same strain collection, whereas peptides expressed by < 5 isolates were classified as “less common.” To analyze the diversity of fHbp across countries and over time, the proportions of isolates harboring prevalent and less common peptides were estimated.

## 3 Results

Of 1,354 identified studies, 52 were included in the review for fHbp data following title and abstract screening.

The included studies were published between 2009 and 2025, representing 34 countries across Asia-Pacific, Europe and Africa, and the Americas. A full list of the included studies, along with the analyzed strain collections, can be found in Supplementary Table S2.

### 3.1 Top fHbp subfamilies by region

Figure 1 presents the latest data for each country reporting fHbp subfamily data; the comprehensive data set (i.e., all studies and time periods for each country) is presented in Supplementary Table S3. Among all included studies, subfamily A variants were reported as dominant in African countries (range, 57.4–67.3% of MenB isolates) and all Asian countries (range, 67.7–98.1%), except for Kazakhstan from 2017 to 2018 and Turkey from 2013 to 2016, where subfamily B dominated [66.7% (*N* = 2 of 3) and 58.3%, respectively]. Subfamily A variants were also reported as dominant in South American countries (range, 62.8–80.0%), except for Brazil during 1988–2006, where subfamily B was dominant (80.0%).

**Figure 1:**
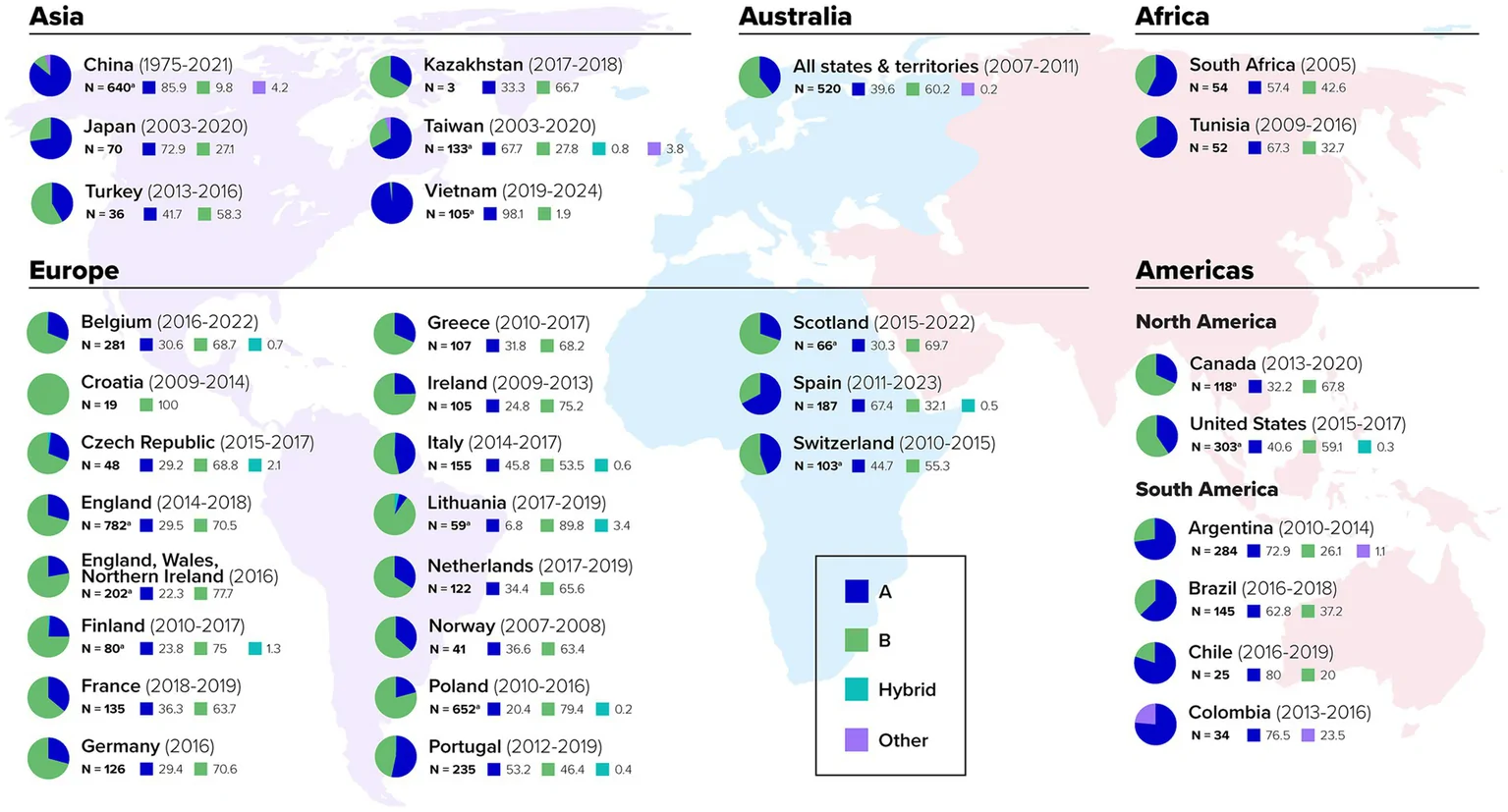
Frequency of reported fHbp subfamilies by country (latest data). fHbp = factor H binding protein; ID = identifier. Subfamily data are presented as percentages. Some studies assessed only a sample of isolates over a given period (Supplementary Table S2). Only the latest available data from included studies are reported here. “Other” includes isolates for which subfamilies were unclear (i.e., data were not reported and could not be determined due to insufficient information on fHbp peptide IDs). Hybrid variants are those that do not fall clearly into either subfamily A or B (i.e., include sequence characteristics of both), as reported by PubMLST (26). ^a^Excluding fHbp-negative strains, those with frameshift or nonsense mutations, and indeterminate isolates.

Subfamily B variants were reported as dominant across all states and territories in Australia from 2007 to 2011 (60.2%), most European countries (range, 52.5–100.0%), except for Portugal during 2012–2019, where subfamily A dominated; Spain in 2009–2010, where subfamilies A and B were in equal proportion; and Spain in 2015–2018 and 2011–2023, where subfamily A dominated), and North America (range, 56.0–67.8%). Among countries with multiple studies covering different time periods, some reports suggest notable variations (i.e., differences >5%) over time. For example, in the Czech Republic, Germany, Italy, and Norway, the proportions of subfamily B variants were reported to decline over time, although subfamily B remained the most common subfamily. In the Netherlands, the reported proportion of subfamily B isolates increased over time, consistently exceeding the proportion of subfamily A isolates.

In certain countries, longitudinal data analysis revealed a shift in the predominant fHbp subfamily over time. In Brazil, subfamily B isolates were dominant in a study of 1988–2006 data, but the proportion was lower in a study of 2016–2018 data, in which subfamily A isolates dominated. Similarly, in Spain, subfamily B isolates were dominant in a study of 2001–2006 data, but their proportion declined across later studies until subfamily A isolates became dominant in studies covering 2015–2023.

### 3.2 Top fHbp peptides by region

#### 3.2.1 Asia-Pacific

The most prevalently reported peptides varied across Asia, with peptide 16 (A19) (29.7%) being most prevalent in a 1975–2021 study in China, peptide 18 (A*) (32.9%) in a 2003–2020 study in Japan, peptides 16 (A19) and 101 (A32) (each, 18.0%) in a 2003–2020 study in Taiwan, and peptide 19 (A22) (59.0%) in a 2019–2024 study in Vietnam (Table 1). Peptide 706 (B*) was most prevalent in Kazakhstan in a 2017–2018 study (66.7%); however, this finding was based on a small sample size (*N* = 3). In Australia, peptide 4 (B16) (14.8%) was the most prevalently reported peptide in a 2007–2011 study of all states and territories. Peptides 1 (B24), 14 (B03), and 16 (A19) were the most prevalently reported peptides in Turkey during 2013–2016 (each, 13.9%).

**Table 1:** Frequency of reported fHbp peptides by country (latest data).

Country (years)	Total isolates, *n*	Unique peptides, *n*	List of identified peptides, PubMLST peptide number/Pfizer variant (%)
Asia			
China (1975–2021)	640^a^	>53^b^	16/A19 (29.7%); 18/A* (11.7%); 404/A* (8.4%); 21/A07 (6.6%); 19/A22 (5.8%); 22/A10 (5.2%); 101/A32 (4.5%); 24/A12 (2.7%); 13/B09 (2.2%); 5/B22 (1.6%); 94/A103, 571/B* (ea., 1.4%); 23/A26 (1.3%); 14/B03 (0.9%); 31/A47, 285/A*, 585/A* (ea., 0.8%); 80/B*, 572/A*, 574/B*, 1,239/A* (ea., 0.6%); 204/A50, 420/B*, 746/A*, 1,028/A*, 1,393/A* (ea., 0.5%); 12/B06, 17/A18, 25/A15, 38/B*, 326/A*, 423/A*, 538/A* (ea., 0.3%); 34/A81, 45/A05, 106/A20, 151/A24, 275/B*, 286/A*, 312/B*, 417/B*, 480/B117, 512/A*, 573/A*, 576/B*, 577/A*, 578/B*, 749/B*, 756/B*, 849/A*, 1,237/A*, 1,240/A*, 1,396/B* (ea., 0.2%); other peptides (total, 4.2%)^c^
Japan (2003–2020)	70	19	18/A* (32.9%); 19/A22 (25.7%); 420/B* (8.6%); 456/B* (5.7%); 4/B16, 12/B06, 16/A19, 21/A07 (ea., 2.9%); 1/B24, 13/B09, 14/B03, 15/B44, 24/A12, 25/A15, 45/A05, 99/A44, 101/A32, 119/A95, 1,149/B* (ea., 1.4%)
Kazakhstan (2017–2018)	3	2	706/B* (66.7%); 849/A* (33.3%)
Taiwan (2003–2020)	133^a^	>27^b^	16/A19, 101/A32 (ea., 18.0%); 19/A22 (12.8%); 13/B09 (12.0%); 276/B153 (6.0%); 420/B* (5.3%); 21/A07, 405/A* (ea., 3.8%); 22/A10, 31/A47, 284/A* (ea., 1.5%); 1/B24, 4/B16, 14/B03, 18/A*, 45/A05, 90/B79, 106/A20, 109/A39, 118/A25, 260/B08, 283/hybrid*, 404/A*, 488/A92, 743/B*, 965/A*, 1,326/A* (ea., 0.8%); other peptides (total, 3.8%)^c^
Turkey (2013–2016)	36	13	**1/B24, 14/B03, 16/A19 (ea., 13.9%)**; 15/B44, 19/A22, 21/A07, 37/B82 (ea., 8.3%); 13/B09, 22/A10, 215/B62 (ea., 5.6%); 4/B16, 47/A06, 1,257/A* (ea., 2.8%)
Vietnam (2019–2024)	105^a^	>5^b^	**19/A22 (59.0%); 101/A32 (24.8%); 106/A20 (7.6%); 21/A07 (4.8%)**; 151/A24 (1.9%); other peptides (total, 1.9%)^c^
Australia			
All states and territories (2007–2011)	520	>12^b^	**4/B16 (14.8%); 19/A22 (12.1%); 31/A47 (10.0%); 15/B44 (8.8%); 14/B03 (7.9%); 224/B30 (5.8%); 1/B24 (5.0%); 13/B09 (4.4%); 45/A05 (3.1%); 29/A99 (2.7%); 21/A07, 110/B23 (ea., 1.7%)**; other peptides (total, 21.9%)^c,d^
Africa			
South Africa (2005)	54	>7^b^	**4/B16 (14.8%); 16/A19, 19/A22 (ea., 13.0%); 1/B24 (11.1%);** 13/B09, 14/B03 (ea., 3.7%); 15/B44 (1.9%); other peptides (total, 38.9%)^c^
Tunisia (2009–2016)	52	16	**47/A06 (25.0%); 13/B09, 19/A22 (ea., 15.4%)**; 16/A19, 119/A95 (ea., 7.7%); 14/B03, 25/A15, 225/B56, 456/B* (ea., 3.8%); 1/B24, 4/B16, 21/A07, 31/A47, 45/A05, 160/A28, 390/B* (ea., 1.9%)
Europe			
Belgium (2016–2022)	281	53	**45/A05 (4.3%)**; other peptides (total, 95.7%)^c^
Croatia (2009–2014)	19	6	**4/B16 (31.6%); 510/B133 (26.3%)**; 10/B64 (21.1%); 14/B03 (10.5%); 1/B24, 249/B41 (ea., 5.3%)
Czech Republic (2015–2017)	48	17	**1/B24 (29.2%); 15/B44 (14.6%); 37/B82 (10.4%)**; 19/A22 (8.3%); 13/B09, 16/A19 (ea., 6.3%); 4/B16 (4.2%); 14/B03, 21/A07, 24/A12, 25/A15, 59/A46, 102/A73, 207/hybrid-A62, 296/A183, 931/B*, 1,114/A* (ea., 2.1%)
England (2014–2018)	782^a^	115	**4/B16 (25.1%); 13/B09 (13.3%); 1/B24 (7.3%); 15/B44 (6.9%); 19/A22 (6.6%); 14/B03 (5.6%); 45/A05 (4.6%); 47/A06 (2.4%); 16/A19 (2.2%); 21/A07 (1.8%); 24/A12 (1.4%); 510/B133 (1.0%); 31/A47 (0.9%); 25/A15, 30/A29 (ea., 0.8%); 69/B78, 86/B83, 110/B23, 483/B119 (ea., 0.6%)**; 37/B82, 61/B* (ea., 0.5%); 215/B62, 218/B47, 224/B30, 364/A*, 499/A108 (ea., 0.4%); 2/B42, 22/A10, 85/A*, 90/B79, 143/A*, 185/A69, 249/B41, 297/B159, 316/A*, 318/B*, 321/B130, 456/B*, 494/A94, 555/A*, 622/B*, 683/A*, 740/A*, 955/A*, 979/B* (ea., 0.3%); 59/A46, 62/B*, 83/A*, 84/A63, 95/A110, 100/B134, 101/A32, 102/A73, 106/A20, 109/A39, 125/B60, 141/A133, 144/B57, 152/A*, 160/A28, 170/A77, 186/A38, 202/A75, 210/B107, 220/B101, 236/B61, 245/B106, 256/B35, 257/B85, 260/B08, 276/B153, 291/B154, 294/A*, 302/A*, 309/B*, 327/B160, 339/A*, 341/A*, 354/A*, 390/B*, 401/A*, 536/A*, 558/A*, 650/B*, 685/A*, 700/B*, 737/A*, 785/B*, 822/B*, 825/B*, 887/B*, 888/A*, 889/B*, 890/B*, 891/A*, 894/A*, 930/A*, 951/B*, 952/A*, 953/A*, 954/B*, 956/A*, 1,059/B*, 1,064/A*, 1,082/A*, 1,150/B*, 1,157/B*, 1,160/B*, 1,161/B*, 1,165/A*, 1,166/A*, 1,217/A*, 1,322/B*, 1,323/A*, 1,502/B* (ea., 0.1%)
England, Wales, Northern Ireland (2016)	202^a^	46	**4/B16 (21.8%); 13/B09 (15.3%); 15/B44 (8.9%); 1/B24 (8.4%); 14/B03 (7.4%); 19/A22 (5.0%); 45/A05 (3.0%)**; 16/A19, 47/A06 (ea., 2.0%); 21/A07, 31/A47, 86/B83, 110/B23, 510/B133 (ea., 1.5%); 30/A29, 90/B79, 215/B62, 224/B30, 321/B130, 979/B* (ea., 1.0%); 2/B42, 22/A10, 25/A15, 37/B82, 62/B*, 102/A73, 143/A*, 186/A38, 200/A74, 257/B85, 260/B08, 276/B153, 297/B159, 302/A*, 341/A*, 456/B*, 622/B*, 683/A*, 738/B*, 785/B*, 951/B*, 952/A*, 953/A*, 954/B*, 955/A*, 956/A* (ea., 0.5%)
Finland (2010–2017)	80^a^	20	**14/B03 (33.8%); 4/B16, 13/B09 (ea., 13.8%); 19/A22 (7.5%); 45/A05 (6.3%)**; 15/B44, 31/A47 (ea., 3.8%); 24/A12 (2.5%); 1/B24, 2/B42, 8/B05, 106/A20, 110/B23, 135/B*, 187/A56, 207/hybrid-A62, 260/B08, 525/B*, 882/A*, 883/B* (ea., 1.3%)
France (2018–2019)	135	30	**510/B133 (29.6%); 45/A05 (9.6%); 47/A06 (7.4%); 19/A22 (6.7%); 14/B03 (5.9%); 13/B09 (5.2%); 15/B44 (4.4%); 4/B16, 321/B130 (ea., 3.7%)**; 1/B24, 21/A07 (ea., 3.0%); other peptides (total, 17.8%)^c^
Germany (2016)	126	32	**14/B03 (15.9%); 1/B24 (11.9%); 45/A05 (10.3%); 15/B44 (9.5%); 4/B16, 19/A22 (ea., 7.9%); 13/B09 (7.1%); 510/B133 (4.0%)**; 110/B23, 232/B74 (ea., 3.2%); 144/B57, 197/A78 (ea., 1.6%); 16/A19, 21/A07, 22/A10, 23/A26, 24/A12, 25/A15, 31/A47, 37/B82, 47/A06, 70/A*, 119/A95, 245/B106, 294/A*, 321/B130, 322/B256, 323/B*, 390/B*, 474/B114, 608/B*, 1,468/A* (ea., 0.8%)
Greece (2010–2017)	107	>21^b^	**1/B24 (24.3%); 15/B44 (14.0%); 14/B03 (11.2%)**; other peptides (total, 50.5%)^c^
Ireland (2009–2013)	105	23	**4/B16 (28.6%); 15/B44 (14.3%); 14/B03 (12.4%); 13/B09 (10.5%); 19/A22 (6.7%); 16/A19 (5.7%)**; 1/B24 (2.9%); other peptides (total, 19.0%)
Italy (2014–2017)	155	43	390/B* (17.4%); 45/A05 (9.0%); 14/B03 (7.1%); 19/A22, 47/A06 (ea., 6.5%); 1/B24 (5.8%); 15/B44, 16/A19 (ea., 4.5%); 13/B09 (3.9%); 4/B16, 119/A95, 160/A28 (ea., 2.6%); 21/A07, 510/B133 (ea., 1.9%); 22/A10, 24/A12, 110/B23, 174/A65, 197/A78, 260/B08, unknown ID/B166 (ea., 1.3%); 2/B42, 25/A15, 31/A47, 59/A46, 94/A103, 100/B134, 185/A69, 224/B30, 245/B106, 291/B154, 296/A183, 297/B159, 322/B256, 362/B258, 1,008/A180, 1,071/B255, 1,078/A179, 1,130/A181, 1,131/A182, 1,132/B257, unknown ID/A184, unknown ID/hybrid* (ea., 0.6%)
Lithuania (2017–2019)	59^a^	11	**1/B24 (79.7%)**; 37/B82, 207/hybrid-A62 (ea., 3.4%); 4/B16, 13/B09, 14/B03, 19/A22, 119/A95, 180/A04, 321/B130, 647/A* (ea., 1.7%)
The Netherlands (2017–2019)	122	36	**1/B24 (16.4%); 14/B03 (14.8%); 510/B133 (12.3%); 19/A22 (9.0%); 15/B44 (8.2%); 45/A05 (7.4%)**; 13/B09, 24/A12 (ea., 3.3%); 123/B91 (2.5%); 144/B57 (1.6%); 4/B16, 16/A19, 21/A07, 31/A47, 47/A06, 69/B78, 96/A*, 100/B134, 191/A72, 294/A*, 316/A*, 333/A*, 390/B*, 476/A85, 486/A91, 494/A94, 544/B*, 581/A*, 603/A*, 627/A*, 715/A*, 1,245/A*, 1,248/A*, 1,249/B*, 1,252/B*, 1,253/B* (ea., 0.8%)
Norway (2007–2008)	41	13	**4/B16 (24.4%); 14/B03 (22.0%); 1/B24, 19/A22 (ea., 12.2%)**; 109/A39 (7.3%); 21/A07 (4.9%); 13/B09, 134/A*, 135/B*, 160/A28, 169/A70, 394/A*, 612/A* (ea., 2.4%)
Poland (2010–2016)	652^a^	74	1/B24 (24.2%); 37/B82 (13.3%); 4/B16 (7.1%); 14/B03 (6.9%); 144/B57 (6.3%); 321/B130 (5.8%); 45/A05 (4.4%); 19/A22 (3.4%); 13/B09 (2.9%); 15/B44 (2.5%); 260/B08 (2.1%); 16/A19 (2.0%); 24/A12, 333/A* (ea., 1.2%); 21/A07, 245/B106 (ea., 1.1%); 584/A* (0.9%); 510/B133 (0.8%); 69/B78, 593/B* (ea., 0.6%); 22/A10, 25/A15, 47/A06, 119/A95, 234/B112, 539/B*, 801/A* (ea., 0.5%); 2/B42, 213/B72, 232/B74, 296/A183, 678/A*, 805/A*, 960/B* (ea., 0.3%); 31/A47, 34/A81, 66/B111, 83/A*, 94/A103, 104/A100, 106/A20, 158/B*, 180/A04, 207/hybrid-A62, 249/B41, 297/B159, 300/A*, 393/B*, 400/A*, 450/B*, 456/B*, 541/A*, 585/A*, 590/B*, 591/B*, 592/B*, 594/B*, 662/A*, 663/A*, 664/B*, 665/B*, 711/A*, 712/B*, 735/A*, 787/A*, 804/A*, 806/B*, 872/B*, 914/B*, 924/A*, 925/B*, 959/B*, 1,087/B*, 1,090/A* (ea., 0.2%)
Portugal (2012–2019)	235	>9^b^	**19/A22 (10.6%); 13/B09 (7.7%); 14/B03 (7.2%); 45/A05 (6.4%); 21/A07 (5.5%); 29/A99 (3.8%); 1/B24, 25/A15 (ea., 3.0%)**; 207/hybrid-A62; other peptides (total, 52.3%)^c^
Spain (2011–2023)	187	68	**45/A05 (19.8%); 1/B24 (8.0%); 47/A06 (7.5%); 19/A22 (7.0%); 16/A19 (4.3%); 13/B09, 25/A15, 260/B08, 494/A94 (ea., 2.7%)**; 14/B03, 21/A07, 510/B133 (ea., 2.1%); 31/A47, 160/A28, 390/B* (ea., 1.6%); 15/B44, 29/A99, 145/B*, 366/A101, 1,292/A*, 1,422/B* (ea., 1.1%); 10/B64, 24/A12, 30/A29, 59/A46, 69/B78, 82/A*, 85/A*, 89/B36, 90/B79, 119/A95, 185/A69, 187/A56, 190/A79, 215/B62, 249/B41, 256/B35, 275/B*, 276/B153, 357/B123, 400/A*, 403/B*, 409/A*, 697/B*, 714/B*, 930/A*, 987/B*, 1,008/A180, 1,010/A*, 1,091/B*, 1,145/B*, 1,199/A*, 1,207/A*, 1,251/A*, 1,254/A*, 1,328/A*, 1,339/A*, 1,341/A*, 1,344/B*, 1,353/A*, 1,354/A*, 1,409/A*, 1,413/A*, 1,414/A*, 1,418/A*, 1,420/A*, 1,421/B*, 1,423/hybrid* (ea., 0.5%)
Switzerland (2010–2015)	103^a^	27	**45/A05 (16.5%); 1/B24, 13/B09, 19/A22 (ea., 9.7%); 4/B16, 14/B03 (ea., 8.7%)**; 197/A78, 510/B133 (ea., 3.9%); 10/B64, 16/A19, 24/A12 (ea., 2.9%); 15/B44, 25/A15, 119/A95, 144/B57, 252/B15 (ea., 1.9%); 21/A07, 22/A10, 83/A*, 224/B30, 291/B154, 297/B159, 527/A*, 1,056/B*, 1,207/A*, 1,312/B*, 1,313/B* (ea., 1.0%)
Americas			
North America			
Canada (2013–2020)	118^a^	23	**15/B44 (29.7%); 19/A22 (18.6%); 4/B16 (16.9%); 13/B09 (10.2%); 45/A05 (4.2%)**; 16/A19, 24/A12 (ea., 2.5%); 1/B24, 210/B107 (ea., 1.7%); 14/B03, 22/A10, 25/A15, 69/B78, 89/B36, 110/B23, 316/A*, 322/B256, 456/B*, 509/B132, 656/A*, 1,134/B*, 1,326/A*, 1,367/B* (ea., 0.8%)
US (2015–2017)	303^a^	53	1/B24 (27.7%); 19/A22 (14.5%); 4/B16 (6.3%); 13/B09 (5.6%); 16/A19 (5.0%); 435/B* (4.6%); 24/A12 (3.6%); 45/A05 (3.0%); 510/B133 (2.3%); 15/B44, 31/A47 (ea., 2.0%); 21/A07, 23/A26, 25/A15 (ea., 1.7%); 14/B03, 18/A*, 89/B36 (ea., 1.3%); 106/A20 (1.0%); 47/A06, 94/A103, 110/B23, 144/B57, 152/A*, 1,121/A* (ea., 0.7%); 27/A106, 49/A17, 100/B134, 102/A73, 125/B60, 157/B*, 224/B30, 233/B98, 235/B70, 243/B53, 261/B104, 276/B153, 297/B159, 339/A*, 420/B*, 430/B*, 509/B132, 569/hybrid*, 592/B*, 677/B*, 814/A*, 888/A*, 904/B*, 972/B*, 975/B*, 984/A*, 1,024/B*, 1,117/B*, 1,119/A* (ea., 0.3%)
South America			
Argentina (2010–2014)	284	>11^b^	16/A19 (15.5%); 119/A95 (11.3%); 19/A22 (6.7%); 47/A06 (6.0%); 658/A* (5.6%); 203/A64 (3.5%); 1/B24, 15/B44, 1,008/A180 (ea., 2.5%); 29/A99, 144/B57 (ea., 2.1%); other peptides (total, 39.8%)^c^
Brazil (2016–2018)	145	34	**24/A12 (18.6%); 47/A06 (16.6%); 1/B24 (14.5%); 13/B09 (8.3%); 45/A05 (6.9%); 4/B16, 119/A95 (ea., 5.5%); 21/A07 (3.4%)**; 499/A108 (2.1%); 31/A47, 275/B* (ea., 1.4%); 14/B03, 15/B44, 16/A19, 19/A22, 25/A15, 62/B*, 161/A*, 174/A65, 178/A52, 187/A56, 218/B47, 226/B95, 346/A*, 544/B*, 552/A*, 740/A*, 1,220/B*, 1,221/B*, 1,230/B*, 1,231/A*, 1,232/A*, 1,233/B*, 1,234/B* (ea., 0.7%)
Chile (2016–2019)	25	12	**19/A22 (48.0%);** 22/A10, 45/A05 (ea., 8.0%); 1/B24, 15/B44, 25/A15, 31/A47, 47/A06, 310/A*, 340/B*, 357/B123, 1,274/B* (ea., 4.0%)
Colombia (2013–2016)	34	8	24/A12 (41.2%); 1,187/A* (26.5%); other peptides (total, 32.4%)^c^

*For peptides without a designated Pfizer variant, subfamilies have been listed. ea. = each; ID = identification number; US = United States. Some studies assessed only a sample of isolates over a given period (Supplementary Table S2). Only the latest available data from included studies are reported here. The fHbp peptides with no Pfizer variant are listed with the subfamily followed only by an asterisk (i.e., A*, B*, hybrid*). Hybrid variants are those that do not fall clearly into either subfamily A or B (i.e., include sequence characteristics of both) as reported by PubMLST (26). Those with only a Pfizer variant are listed as “Unknown ID.” Unless specified otherwise, bolding denotes prevalent peptides (i.e., those that were expressed by ≥5 isolates). For Kazakhstan, as there were three isolates in total, no peptides qualified as “prevalent” and therefore no peptides are bolded in the table. ^a^Excluding fHbp-negative strains, those with frameshift or nonsense mutations, and indeterminate isolates. ^b^Based on the available data. The exact number was not specified. ^c^Includes other peptides that were not delineated in the source or did not have an associated frequency reported, including less common and/or novel peptides. ^d^Includes 15 strains with incomplete sequencing. These were not excluded, as subfamily details were available.

#### 3.2.2 Europe and Africa

Peptides 1 (B24), 4 (B16), and 14 (B03) were commonly reported as the most prevalent fHbp peptides across European countries, though variations over time and between countries were observed (Table 1). In the Czech Republic, peptides 1 (B24) and 37 (B82) (each, 17.9%) were reported as the most prevalent during 2001–2006, and peptide 1 (B24) (29.2%) was most prevalent during 2015–2017. Peptide 1 (B24) was the most prevalently reported peptide in Lithuania during 2017–2019 (79.7%) and in Poland during 2010–2016 (24.2%).

In France, peptide 14 (B03) and peptide 1 (B24) were the 2 most prevalently reported peptides in 2001–2006 (22.5 and 19.7%, respectively), 2007–2008 (19.0 and 21.5%, respectively), and 2013–2014 (14.6 and 15.8%, respectively). Notably, peptide 510 (B133) emerged as the most prevalently reported peptide in the latest isolate collection analyzed (2018–2019; 29.6%). Peptide 1 (B24) was the most prevalently reported peptide in Germany in a 2001–2006 study (27.8%), followed by peptide 14 (B03) (18.5%). In a subsequent data set, however, the prevalence of peptide 1 (B24) was remarkably lower, with peptide 14 (B03) predominating in 2007–2008 (21.2%) and 2016 (15.9%) reports. Studies in the Netherlands indicated that peptide 14 (B03) (22.5%) was most prevalent during 1960–2009, followed by peptide 16 (A19) (15.8%) and peptide 1 (B24) (10.8%), whereas during 2017–2019, peptide 1 (B24) (16.4%) became the most prevalent peptide and the prevalence of peptide 14 (B03) (14.8%) decreased. In Switzerland, the most prevalent peptide reported in a 2010–2015 study was peptide 45 (A05) (16.5%).

Peptide 4 (B16) was consistently reported as the most prevalent peptide in the United Kingdom (UK) across MenB isolates in studies spanning 2001–2006 (England, Wales, Northern Ireland; 23.5%), 2007–2008 (England, Wales; 22.5%), 2014–2015 (England, Wales, Northern Ireland; 20.3%), 2016 (England, Wales, Northern Ireland; 21.8%), and 2014–2018 (England; 25.1%). Peptide 4 (B16) was also the most prevalently reported peptide in a 2009–2013 study in Ireland (28.6%). In Norway, peptide 1 (B24) was exclusively detected during 1985–1990 (100%) due to a historic epidemic but persisted at lower rates in 2001–2006 (34.8%) and 2005–2006 (26.5%). In contrast, peptide 4 (B16) was the most prevalently reported peptide in a 2007–2008 study (24.4%). Peptide 14 (B03) was the most prevalently reported peptide in a 2010–2017 study in Finland (33.8%).

In Spain, peptide 1 (B24) was the most prevalently reported peptide in a strain collection covering 2001–2006 (26.6%) and 2009–2010 (19.3%), with peptide 45 (A05) becoming the most prevalent in studies covering 2015–2018 (16.1%) and 2011–2023 (19.8%). Studies in Greece identified peptide 15 (B44) (27.7%) as the most prevalent peptide during 1999–2010 and peptide 1 (B24) (24.3%) as the most prevalent peptide during 2010–2017. In Italy, peptide 14 (B03) (31.5%) was the most prevalent peptide during 2007–2008, whereas its occurrence was remarkably lower in an isolate collection in 2014–2017, when peptide 390 (B*) (17.4%) was most prevalent. Peptide 4 was the most prevalently reported peptide in Croatia in a 2009–2014 study (31.6%), and peptide 19 (A22) was the most prevalently reported peptide in Portugal in a 2012–2019 study (10.6%).

The most prevalently reported peptide in a 2005 study in South Africa was peptide 4 (B16) (14.8%), and peptide 47 (A06) was the most prevalently reported peptide in Tunisia in a 2009–2016 study (25.0%).

#### 3.2.3 The Americas

Peptide 15 (B44) was the most prevalently reported fHbp peptide in Canada during 2006–2012 (32.7%) and 2013–2020 (29.7%) (Table 1). In studies from the United States (US), peptide 1 (B24) was consistently reported as the most prevalent peptide in studies spanning 2001–2006 (29.6%), 2000–2008 (32.6%), 2009–2014 (34.0%), and 2015–2017 (27.7%).

The most prevalently reported peptides varied across South American countries, with peptide 16 (A19) (15.5%) being most prevalent in a 2010–2014 study in Argentina; peptide 1 (B24) (67.0%) in a 1988–2006 study in Brazil, which ranked among the 3 most prevalent peptides in a 2016–2018 study in Brazil [peptide 24 (A12) (18.6%), peptide 47 (A06) (16.6%), and peptide 1 (B24) (14.5%)]; peptide 19 (A22) (48.0%) in a 2016–2019 study in Chile; and peptide 24 (A12) (41.2%) in a 2013–2016 study in Colombia.

#### 3.2.4 Overall diversity

Our analysis revealed that less common fHbp peptides (i.e., those expressed by < 5 isolates in a study) accounted for a considerable proportion of reported cases in studies in some countries. For example, in a 2011–2023 study in Spain (*N* = 187; 68 unique peptides), 42.8% of cases were caused by isolates expressing 59 less common peptides, whereas the remaining 57.2% of isolates expressed 9 prevalent peptides (Table 1). In a 2017–2019 panel in the Netherlands (*N* = 122; 36 unique peptides), 32.0% of reported cases were caused by isolates expressing 30 less common peptides. In a UK panel of isolates from 2016 (*N* = 202; 46 unique peptides), 7 fHbp peptides were expressed by 69.8% of isolates and the remaining isolates expressed 39 different variants. Although to a lesser extent, a US panel of isolates collected during 2015–2017 (*N* = 303; 53 unique peptides) also demonstrated a notable portion of less common peptides, with isolates from 18.5% of cases reported to express less common peptides. In contrast, lower fHbp diversity was observed in some countries such as Vietnam (2019–2024), where less common peptides accounted for only 3.8% of reported peptides.

## 4 Discussion

This global targeted literature review focused on the frequency and diversity of reported fHbp subfamilies and peptides for MenB isolates, expanding understanding of current disease trends. The 52 included studies across 34 countries showed that MenB-fHbp subfamily A variants were typically dominant in reports from Africa, Asia, and South America, and subfamily B variants were typically dominant in reports from Australia, Europe, and North America. Some regions had relatively stable trends over time (e.g., the UK), whereas others had more temporal diversity (e.g., Spain). Prevalently reported MenB-fHbp peptides varied between countries and regions as well as within countries over time; understanding this diversity is important to elucidate the likely coverage of MenB vaccines.

Previous studies have suggested that fHbp is important for the survival of pathogenic *N. meningitidis in vivo*, which, in addition to its immunogenic properties, likely creates selective pressure and drives diversity (21, 27–29). Accordingly, the results of this study highlight the diversity and unpredictability of MenB strains, with the most prominent fHbp peptides varying between countries and over time and a multitude of less common peptides circulating. For instance, in a 2015–2017 US study (*N* = 303), 18.5% of disease isolates expressed less common fHbp peptides (i.e., those expressed by < 5 isolates), with a total of 53 different peptide variants reported (30). Furthermore, in a 2011–2023 study in Spain (*N* = 187), 42.8% of cases were caused by strains expressing less common fHbp peptides, with a total of 68 different peptide variants reported (31). Given the diversity of fHbp in MenB isolates identified in this global review, it is important to continue to monitor the regional epidemiology of fHbp.

Furthermore, epidemiological fluctuations in MenB-fHbp data over time were observed across studies in some countries included in this review, in line with previous reports (32, 33). However, the possibility cannot be excluded that the rollout of MenB national vaccination programs or private vaccination uptake in some countries between studies conducted at different time points could have influenced the observed fHbp peptide epidemiology of disease-causing strains over time. Nonetheless, temporal variations in peptide epidemiology might highlight the importance of implementing vaccines with broad coverage within routine immunization programs as well as for the control of outbreaks, such as those observed in universities and nurseries, which, depending on the strain causing disease, suggest the potential for improved coverage with MenB-fHbp versus 4CMenB vaccination (24, 25, 34–37).

To the best of our knowledge, this is the first review to explore both the reported prevalence and diversity of MenB-fHbp across the globe. A strength of this study is that no search limitations were applied, allowing for the inclusion of a broad range of studies across countries and time periods. However, this study is subject to limitations. The results and descriptive comparisons should be interpreted cautiously, as studies differed in key factors such as design, data collection periods, sample sizes, and reporting standards. In addition, this was a targeted review, and gray literature data were not reviewed. As data were not available from all countries at all time points and some sources only included a sample of isolates, the results may not be fully representative of all countries and time periods. Furthermore, the majority of the reports did not stratify data by age (38), which is known to influence fHbp distribution (27, 39). Molecular data were collected from isolates; however, fHbp expression data, which are important for predicting the breadth of vaccine coverage, were not reviewed (40). It should be noted that data from isolates may fundamentally differ from data from cases for which no isolates were collected (i.e., cases confirmed using only nonculture clinical specimens) (41). The data reported in this study relate to MenB only and, therefore, may not be applicable to fHbp from other serogroups. As this review evaluated fHbp diversity in invasive MenB isolates only, the findings cannot be extrapolated to other *Neisseria* species (e.g., *N. gonorrhoeae*). Finally, as the PubMed search was conducted in June 2025, fHbp variants associated with more recent outbreaks, such as the UK 2026 outbreak (42), are not accounted for in the results.

Future studies using 100% whole-genome sequencing and open sharing (e.g., via platforms such as the Global Meningitis Genome Library, available through PubMLST) can make further analysis, global collaboration, and real-time monitoring of circulating strains possible (43, 44). Such sequencing efforts, combined with phylogenetic analyses, could provide additional insight into the temporal and geographic changes described in this review.

## 5 Conclusion

The results of this review demonstrate the diversity of fHbp in MenB isolates in 34 countries across the globe. The observed variations in MenB-fHbp epidemiology between countries and regions and at different time points within countries highlight the importance of continued surveillance in multiple countries and over time. The continual and unpredictable evolution of fHbp diversity and frequency underscores the need for vaccines with broad fHbp coverage.
