# Supplementary material for: Global epidemiology of the factor H binding protein in meningococcal serogroup B invasive isolates: a targeted literature review
Source: Front Public Health. 2026 Oct 12;14:1937472. doi: 10.3389/fpubh.2026.1937472 (PMC13696914; doi:10.3389/fpubh.2026.1937472)
Supplement: Supplementary file 1 [file Data_Sheet_1.pdf]

Supplementary Material

Table S1. PubMed Search Terms

Query	Search details	Results
**Keywords**		
fhbp	"fhbp"[All Fields]	418
factor h binding protein	"factor h binding protein"[Text Word]	357
(Meningococcal Serogroup B) AND (invasive isolates) AND (epidemiology)	("meningococcal"[All Fields] OR "meningococcic"[All Fields]) AND ("serogroup"[MeSH Terms] OR "serogroup"[All Fields] OR "serogrouping"[All Fields] OR "serogroups"[All Fields] OR "serogroupable"[All Fields] OR "serogrouped"[All Fields]) AND "B"[All Fields] AND (("invasibility"[All Fields] OR "invasible"[All Fields] OR "invasion"[All Fields] OR "invasions"[All Fields] OR "invasive"[All Fields] OR "invasively"[All Fields] OR "invasiveness"[All Fields] OR "invasives"[All Fields] OR "invasivity"[All Fields]) AND ("isolate"[All Fields] OR "isolate s"[All Fields] OR "isolated"[All Fields] OR "isolates"[All Fields] OR "isolating"[All Fields] OR "isolation and purification"[MeSH Subheading] OR ("isolation"[All Fields] AND "purification"[All Fields]) OR "isolation and purification"[All Fields] OR "isolation"[All Fields] OR "isolations"[All Fields])) AND ("epidemiologies"[All Fields] OR "epidemiology"[MeSH Subheading] OR "epidemiology"[All Fields] OR "epidemiology"[MeSH Terms] OR "epidemiology s"[All Fields])	312
**MeSH terms**		
"Neisseria meningitidis, Serogroup B/classification"[MeSH]	"neisseria meningitidis, serogroup b/classification"[MeSH Terms]	75
"Neisseria meningitidis, Serogroup B/isolation	"neisseria meningitidis, serogroup b/isolation and purification"[MeSH Major Topic]	77

Query	Search details	Results
and purification”[MAJR]		
“Neisseria meningitidis, Serogroup B/genetics”[MAJR]	“neisseria meningitidis, serogroup b/genetics”[MeSH Major Topic]	115

MeSH = Medical Subject Headings.

Table S2. Included Studies

Region	Study period	Collection details	Citation
**Africa**			
Tunisia	2009-2016	Laboratory of Microbiology at Bechir Hamza Children's Hospital in Tunis (Tunisia)	Brik A, et al. Int J Infect Dis. 2020;91:73-8. doi: 10.1016/j.ijid.2019.11.013.
**Asia**			
China	1975-2021[a]	Chinese Center for Disease Control and Prevention	Tan Z, et al. Microorganisms. 2024;12(3):481. doi: 10.3390/microorganisms12030481.
Japan	2003-2020	Domestic prefectural public health laboratories or hospitals	Takahashi H, et al. Vaccine. 2023;41(2):416-26. doi: 10.1016/j.vaccine.2022.10.083.
Kazakhstan	2017-2018	The State Epidemiological Service	Shevtsov A, et al. PLoS One. 2022;17(12):e0279536. doi: 10.1371/journal.pone.0279536.
Taiwan	2003-2020	Taiwan Centers for Disease Control	Chiou C-S, et al. Microbiol Spectr. 2022;10(4):e0088222. doi: 10.1128/spectrum.00882-22.
Turkey	2013-2017[b]	Department of Pediatric Infectious Diseases, Hacettepe University Faculty of Medicine	Ceyhan M, et al. Vaccine. 2020;38(19):3545-52. doi: 10.1016/j.vaccine.2020.03.024.
Vietnam	2019-2024	Isolates collected in Vietnam	Long TP, et al. Pathogens. 2025;14(5):487. doi: 10.3390/pathogens14050487.
**Australia**			
Australia (Western Australia)	2000-2014[c]	The PathWest reference laboratory	Mowlaboccus S, et al. PLoS One. 2016;11(6):e0158315. doi: 10.1371/journal.pone.0158315.

Region	Study period	Collection details	Citation
Australia (all states and territories)	2007-2011	National Neisseria Network state reference laboratories	Tozer SJ, et al. Hum Vaccin Immunother. 2021;17(9):3230-8. doi: 10.1080/21645515.2021.1904758.
Australia (Western Australia, Victoria)	2008-2012[c]	The National Neisseria Network	Mowlaboccus S, et al. PLoS One. 2017;12(10):e0186839. doi: 10.1371/journal.pone.0186839.
**Europe**			
Belgium	2016-2022	The National Reference Centre	Goeders N, et al. Vaccine. 2025;55:127026. doi: 10.1016/j.vaccine.2025.127026.
Croatia	2009-2014	The Croatian National Institute of Public Health	Bukovski S, et al. J Med Microbiol. 2016;65(9):1013-19. doi: 10.1099/jmm.0.000320.
Czech Republic	2015-2017	National Reference Laboratory for Meningococcal Infections	Krizova P, Honskus M. PLoS One. 2019;14(7):e0219477. doi: 10.1371/journal.pone.0219477.
Finland	2010-2017	National Infectious Disease Register	Bodini M, et al. mSphere. 2020;5(5):e00376-20. doi: 10.1128/mSphere.00376-20.
France, Germany, Italy, Norway, UK (England, Wales)	2007-2008[d]	European Reference Laboratories	Vogel U, et al. Lancet Infect Dis. 2013;13(5):416-25. doi: 10.1016/S1473-3099(13)70006-9.
France	2013-2019	The National Reference Center for Meningococci and *Haemophilus influenzae*	Hong E, et al. Hum Vaccin Immunother. 2021;17(12):5614-22. doi: 10.1080/21645515.2021.2004055.

Region	Study period	Collection details	Citation
Germany, Italy, Spain, UK (England, Wales, Northern Ireland)	2016	National reference laboratories	Clark SA, et al. Hum Vaccin Immunother. 2024;20(1):2427471. doi: 10.1080/21645515.2024.2427471.
Greece	1999-2010[e]	The National Meningitis Reference Laboratory at the National School of Public Health	Tzanakaki G, et al. BMC Microbiol. 2014;14:111. doi: 10.1186/1471-2180-14-111.
Greece	2010-2017	The National Public Health Organization	Tzanakaki G, et al. Vaccine. 2021;39(11):1621-30. doi: 10.1016/j.vaccine.2021.01.073.
Italy (Tuscany)	2007-2019[c]	Molecular Surveillance Registry	Lodi L, et al. J Infect. 2021;82(4):28-36. doi: 10.1016/j.jinf.2020.12.034.
Italy	2014-2017	National Surveillance System of Invasive Meningococcal Disease	Carannante A, et al. PLoS One. 2020;15(11):e0241793. doi: 10.1371/journal.pone.0241793.
Lithuania	2017-2019	The National Public Health Surveillance Laboratory	Sereikaitė E, et al. Front Cell Infect Microbiol. 2023;13:1136211. doi: 10.3389/fcimb.2023.1136211.
The Netherlands	1960-2009[f]	The Netherlands Reference Laboratory for Bacterial Meningitis	Bambini S, et al. PLoS One. 2013;8(5):e65043. doi: 10.1371/journal.pone.0065043.
The Netherlands	2017-2019	The Reference Laboratory for Bacterial Meningitis	Freudenburg-de Graaf W, et al. Vaccine. 2020;38(49):7850-7. doi: 10.1016/j.vaccine.2020.10.008.
Norway	1985-1990, 2005-2006	The National Reference Laboratory for Meningococci at the Norwegian Institute of Public Health and the Ullevål University Hospital in Oslo	Holst J, et al. Vaccine. 2014;32(23):2722-31. doi: 10.1016/j.vaccine.2014.02.068.

Region	Study period	Collection details	Citation
Poland	2010-2011[g]	The Polish National Reference Centre for Bacterial Meningitis	Waśko I, et al. Vaccine. 2016;34(4):510-5. doi: 10.1016/j.vaccine.2015.11.070.
Poland	2010-2016	The Polish National Reference Centre for Bacterial Meningitis	Waśko I, et al. Vaccine. 2020;38(8):1943-52. doi: 10.1016/j.vaccine.2020.01.021.
Portugal	2012-2019	National Reference Laboratory for *Neisseria meningitidis* at the Portuguese National Institute of Health	Bettencourt C, et al. Vaccine. 2022;40(33):4772-9. doi: 10.1016/j.vaccine.2022.06.038.
Ireland	2009-2013	Irish Meningitis and Sepsis Reference Laboratory	Mulhall Robert M, et al. mSphere. 2018;3(4):e00196-18. doi: 10.1128/mSphere.00196-18.
Spain	2009-2010[f]	Spanish National Reference Laboratory for Meningococci	Abad R, et al. PLoS One. 2016;11(3):e0150721. doi: 10.1371/journal.pone.0150721.
Spain	2011-2023	Clinical microbiology laboratories	Roca-Grande J, et al. Emerg Microbes Infect. 2025;14(1):2482696. doi: 10.1080/22221751.2025.2482696.
Spain	2015-2018	Spanish National Reference Laboratory	Abad R, et al. J Infect. 2021;82(4):37-44. doi: 10.1016/j.jinf.2021.01.030.
Switzerland	2010-2015	Swiss National Reference Center for Meningococci	Leo S, et al. Vaccine. 2020;38(33):5324-31. doi: 10.1016/j.vaccine.2020.05.071.
UK (England, Wales, Northern Ireland)	2014-2015[d]	Public Health England Meningococcal Reference Unit	Parikh SR, et al. Lancet Infect Dis. 2017;17(7):754-62. doi: 10.1016/S1473-3099(17)30170-6.
UK (England)	2014-2018[f]	Public Health England Meningococcal Reference Unit	Lucidarme J, et al. J Infect. 2022;84(2):136-44. doi: 10.1016/j.jinf.2021.11.015.

Region	Study period	Collection details	Citation
UK (Scotland)	2015-2022[b]	Meningococcal Invasive Disease Augmented Surveillance	Rodrigues CMC, et al. mBio. 2023;14(2):e0049923. doi:10.1128/mbio.00499-23.
**North America**			
Canada	2006-2009[g]	Canadian Immunization Monitoring Program Active surveillance network	Bettinger JA, et al. Vaccine. 2013;32(1):124-30. doi: 10.1016/j.vaccine.2013.03.063.[g]
Canada	2006-2012	Canadian Immunization Monitoring Program Active surveillance network	Bettinger JA, et al. Vaccine. 2020;38(8):2026-33. doi: 10.1016/j.vaccine.2019.12.051.
Canada (Quebec)	2009-2013[c]	National Microbiology Laboratory of the Public Health Agency of Canada	Law DK, et al. BMC Microbiol. 2015;15:143. doi: 10.1186/s12866-015-0469-6.
Canada	2010-2014[g]	National Microbiology Laboratory	Tsang RSW, et al. mSphere. 2020;5(2):e00883-19. doi: 10.1128/mSphere.00883-19.
Canada	2013-2020	Canadian Immunization Monitoring Program Active surveillance network	Meesters K, et al. Vaccine. 2025;55:127030. doi: 10.1016/j.vaccine.2025.127030.
US	2000-2008[h]	Active Bacterial Core surveillance	Rajam G, et al. mSphere. 2017;2(6):e00261-17. doi: 10.1128/mSphere.00261-17.
US	2000-2008[g,h]	Active Bacterial Core surveillance	Wang X, et al. Vaccine. 2011;29(29-30):4739-44. doi: 10.1016/j.vaccine.2011.04.092.
US	2009-2014[h]	Active Bacterial Core surveillance and expanded surveillance sites	Chang H-Y, et al. J Infect. 2019;79(5):426-34. doi: 10.1016/j.jinf.2019.09.001.
US	2015-2017[f]	Active Bacterial Core surveillance	Muzzi A, et al. mSphere. 2022;7(5):e0038522. doi: 10.1128/msphere.00385-22.

Region	Study period	Collection details	Citation
**South America**			
Argentina	2010-2014	Argentinian National Laboratories Network	Efron A, et al. Hum Vaccin Immunother. 2024;20(1):2378537. doi: 10.1080/21645515.2024.2378537.
Brazil	1988-2006[f]	Brazilian National Meningitis Surveillance System	De Filippis I, et al. PLoS One. 2012;7(3):e33016. doi: 10.1371/journal.pone.0033016.
Brazil	2016-2018[f]	Ministry of Health in Brazil	De Lemos APS, et al. PLoS One. 2020;15(12):e0243375. doi: 10.1371/journal.pone.0243375.
Chile	2016-2019[b]	Public Health Institute	Arteta-Acosta C, et al. Vaccine. 2024;42(26):126311. doi: 10.1016/j.vaccine.2024.126311.
Colombia	2013-2016	Colombian Instituto Nacional de Salud	Alarcon ZK, et al. Enferm Infecc Microbiol Clin (Engl Ed). 2023;41(5):290-3. doi: 10.1016/j.eimce.2022.01.005.
**Multicountry**			
Czech Republic, France, Germany, Norway, Spain, US	2001-2006[f,h]	Public Health Laboratories of the Czech Republic, France, Norway, and US; reference laboratories from Germany and Spain	Hoiseth SK, et al. Pediatr Infect Dis J. 2013;32(10):1096-101. doi: 10.1097/INF.0b013e31829aa63b.
Czech Republic, France, Norway, South Africa, UK (England, Wales, Northern Ireland)	2000-2006[f]	Public health laboratories in the Czech Republic, France, Norway, and the US, and from the Health Protection Agency in Manchester for the UK; the Group for Enteric, Respiratory, and Meningeal Disease Surveillance in South Africa	Murphy E, et al. J Infect Dis. 2009;200(3):379-89. doi: 10.1086/600141.

UK, United Kingdom; US = United States.

[a] This study was conducted from 1956 to 2021; however, MenB isolates were available only from 1975.

[b] Only included isolates from children.

[c] Data were regional, so were not summarized.

[d] Epidemiologic year (July to June).

[e] Strains isolated from 1999 to 2007 were enriched for the cc162 and cc269.

[f] A sample of isolates were included.

[g] Reported overlapping data with another study, so were not summarized.

[h] US data were corrected for disease prevalence in Oregon.

Table S3. Frequency of Reported fHbp Subfamilies by Country (All Data)

Region	Study period	Total number of MenB isolates	Subfamily, %				Reference
			A	B	Hybrid	Other	
**Asia**							
China	1975-2021	640[a]	85.9	9.8	0	4.2	Tan et al. (1)
Japan	2003-2020	70	72.9	27.1	0	0	Takahashi et al. (2)
Kazakhstan	2017-2018	3	33.3	66.7	0	0	Shevtsov et al. (3)
Taiwan	2003-2020	133[a]	67.7	27.8	0.8	3.8	Chiou et al. (4)
Turkey	2013-2016	36	41.7	58.3	0	0	Ceyhan et al. (5)
Vietnam	2019-2024	105[a]	98.1	1.9	0	0	Long et al. (6)
**Australia**							
All states and territories	2007-2011	520	39.6	60.2	0	0.2	Tozer et al. (7)
**Africa**							
South Africa	2005	54	57.4	42.6	0	0	Murphy et al. (8)
Tunisia	2009-2016	52	67.3	32.7	0	0	Brik et al. (9)
**Europe**							
Belgium	2016-2022	281	30.6	68.7	0.7	0	Goeders et al. (10)
Croatia	2009-2014	19	0	100.0	0	0	Bukovski et al. (11)
Czech Republic	2001-2006	28	25.0	75.0	0	0	Hoiseth et al. (12)
	2015-2017	48	29.2	68.8	2.1	0	Krizova and Honskus (13)
Finland	2010-2017	80[a]	23.8	75.0	1.3	0	Bodini et al. (14)
France	2001-2006	244	32.4	67.6	0	0	Hoiseth et al. (12)
	2007-2008	200	37.5	62.5	0	0	Vogel et al. (15)
	2013-2014	171	40.9	59.1	0	0	Hong et al. (16)

Region	Study period	Total number of MenB isolates	Subfamily, %				Reference
			A	B	Hybrid	Other	
	2018-2019	135	36.3	63.7	0	0	Hong et al. (16)
Germany	2001-2006	205	21.0	79.0	0	0	Hoiseth et al. (12)
	2007-2008	222	26.1	73.9	0	0	Vogel et al. (15)
	2016	126	29.4	70.6	0	0	Clark et al. (17)
Greece	1999-2010	148	33.1	66.9	0	0	Tzanakaki et al. (18)
	2010-2017	107	31.8	68.2	0	0	Tzanakaki et al. (19)
Ireland	2009-2013	105	24.8	75.2	0	0	Mulhall Robert et al. (20)
Italy	2007-2008	54	37.0	63.0	0	0	Vogel et al. (15)
	2014-2017	155	45.8	53.5	0.6	0	Carannante et al. (21)
Lithuania	2017-2019	59[a]	6.8	89.8	3.4	0	Sereikaitė et al. (22)
The Netherlands	1960-2009	120[a]	46.7	52.5	0.8	0	Bambini et al. (23)
	2017-2019	122	34.4	65.6	0	0	Freudenburg-de Graaf et al. (24)
Norway	1985-1990	28	0	100.0	0	0	Holst et al. (25)
	2001-2006	23	34.8	65.2	0	0	Hoiseth et al. (12)
	2005-2006	49	34.7	65.3	0	0	Holst et al. (25)
	2007-2008	41	36.6	63.4	0	0	Vogel et al. (15)
Poland	2010-2016	652[a]	20.4	79.4	0.2	0	Waśko et al. (26)
Portugal	2012-2019	235	53.2	46.4	0.4	0	Bettencourt et al. (27)
Spain	2001-2006	346	40.2	59.8	0	0	Hoiseth et al. (12)
	2009-2010	300	50.0	50.0	0	0	Abad et al. (28)
	2015-2018	409[a]	66.0	32.5	1.5	0	Abad et al. (29)
	2011-2023	187	67.4	32.1	0.5	0	Roca-Grande et al. (30)
Switzerland	2010-2015	103[a]	44.7	55.3	0	0	Leo et al. (31)

Region	Study period	Total number of MenB isolates	Subfamily, %				Reference
			A	B	Hybrid	Other	
United Kingdom							
England, Wales, Northern Ireland	2001-2006	536	23.3	76.7	0	0	Murphy et al. (8)
England, Wales	2007-2008	534[a]	29.0	71.0	0	0	Vogel et al. (15)
England, Wales, Northern Ireland	2014-2015	251	31.9	68.1	0	0	Parikh et al. (32)
England, Wales, Northern Ireland	2016	202[a]	22.3	77.7	0	0	Clark et al. (17)
England	2014-2018	782[a]	29.5	70.5	0	0	Lucidarme et al. (33)
Scotland	2015-2022	66[a]	30.3	69.7	0	0	Rodrigues et al. (34)
**North America**							
Canada	2006-2012	275[a]	0	63.3	0	36.7	Bettinger et al. (35)
	2013-2020	118[a]	32.2	67.8	0	0	Meesters et al. (36)
United States	2001-2006	273	42.9	57.1	0	0	Hoiseth et al. (12)
	2000-2008	442	40.3	59.7	0	0	Rajam et al. (37)
	2009-2014	100	44.0	56.0	0	0	Chang et al. (38)
	2015-2017	303[a]	40.6	59.1	0.3	0	Muzzi et al. (39)

Region	Study period	Total number of MenB isolates	Subfamily, %				Reference
			A	B	Hybrid	Other	
**South America**							
Argentina	2010-2014	284	72.9	26.1	0	1.1	Efron et al. (40)
Brazil	1988-2006	382	0	80.0	0	20.0	de Filippis et al. (41)
	2016-2018	145	62.8	37.2	0	0	de Lemos et al. (42)
Chile	2016-2019	25	80.0	20.0	0	0	Arteta-Acosta et al. (43)
Colombia	2013-2016	34	76.5	0	0	23.5	Alarcon et al. (44)

fHbp = factor H binding protein; MenB = meningococcal serogroup B.

Notes: Green shading represents the most prevalent subfamily category per row. Some studies assessed only a sample of isolates over a given period (Table S-2). “Other” includes isolates with unspecified results. Hybrid variants are those that do not fall clearly into either subfamily A or B (i.e., include sequence characteristics of both) as reported by PubMLST (45).

[a] Excluding fHbp-negative strains, those with frameshift or nonsense mutations, and indeterminate isolates.

References

1. Tan Z, Xu J, Che J, Xu L, Yan D, Zhang M, et al. Extensive genetic diversity and epidemiological patterns of factor H-binding protein variants among *Neisseria meningitidis* in China. Microorganisms (2024) 12:481.

2. Takahashi H, Morita M, Kamiya H, Fukusumi M, Sunagawa M, Nakamura-Miwa H, et al. Genomic characterization of Japanese meningococcal strains isolated over a 17-year period between 2003 and 2020 in Japan. Vaccine (2023) 41:416–26. doi: https://doi.org/10.1016/j.vaccine.2022.10.083

3. Shevtsov A, Aushakhmetova Z, Amirgazin A, Khegay O, Kamalova D, Sanakulova B, et al. Whole genome sequence analysis of *Neisseria meningitidis* strains circulating in Kazakhstan, 2017–2018. PLoS One (2022) 17:e0279536. doi: 10.1371/journal.pone.0279536

4. Chiou C-S, Liao Y-S, Chen B-H, Lu M-C, Hong Y-P, Wang Y-W, et al. Demographic features of invasive meningococcal disease in Taiwan, 1993 to 2020, and genetic characteristics of *Neisseria meningitidis* isolates, 2003 to 2020. Microbiol Spectr (2022) 10:e00882–22. doi: 10.1128/spectrum.00882-22

5. Ceyhan M, Ozsurekci Y, Lucidarme J, Borrow R. Characterization of invasive *Neisseria meningitidis* isolates recovered from children in Turkey during a period of increased serogroup B disease, 2013–2017. Vaccine (2020) 38:3545–52. doi: https://doi.org/10.1016/j.vaccine.2020.03.024

6. Long TP, Cuong VV, Anh BTL, Van Toan T, Loan VT, Hung PV, et al. Genetic and antigenic diversity of *Neisseria meningitidis* serogroup B strains in Vietnam. Pathogens (2025) 14:487.

7. Tozer SJ, Smith HV, Whiley DM, Borrow R, Boccadifuoco G, Medini D, et al. High coverage of diverse invasive meningococcal serogroup B strains by the 4-component vaccine 4CMenB in Australia, 2007–2011: concordant predictions between MATS and genetic MATS. Hum Vaccin Immunother (2021) 17:3230–8. doi: 10.1080/21645515.2021.1904758

8. Murphy E, Andrew L, Lee K-L, Dilts DA, Nunez L, Fink PS, et al. Sequence diversity of the factor H binding protein vaccine candidate in epidemiologically relevant strains of serogroup B *Neisseria meningitidis*. J Infect Dis (2009) 200:379–89. doi: 10.1086/600141

9. Brik A, Terrade A, Hong E, Deghmane A, Taha MK, Bouafsoun A, et al. Phenotypic and genotypic characterization of meningococcal isolates in Tunis, Tunisia: high diversity and impact on vaccination strategies. Int J Infect Dis (2020) 91:73–8. doi: 10.1016/j.ijid.2019.11.013

10. Goeders N, Vanneste K, Roosens NHC, Bogaerts B, Mattheus W. Molecular and WGS-based characterization of invasive *Neisseria meningitidis* isolates collected in Belgium (2016-2022) and MenB-FHbp vaccine coverage estimation of serogroup B. Vaccine (2025) 55:127026. doi: 10.1016/j.vaccine.2025.127026

11. Bukovski S, Vacca P, Anselmo A, Knezovic I, Fazio C, Neri A, et al. Molecular characterization of a collection of *Neisseria meningitidis* isolates from Croatia, June 2009 to January 2014. J Med Microbiol (2016) 65:1013–9. doi: 10.1099/jmm.0.000320

12. Hoiseth SK, Murphy E, Andrew L, Vogel U, Frosch M, Hellenbrand W, et al. A multi-country evaluation of *Neisseria meningitidis* serogroup B factor H–binding proteins and implications for vaccine coverage in different age groups. Pediatr Infect Dis J (2013) 32:1096–101. doi: 10.1097/INF.0b013e31829aa63b

13. Krizova P, Honskus M. Genomic surveillance of invasive meningococcal disease in the Czech Republic, 2015-2017. PLoS One (2019) 14:e0219477. doi: 10.1371/journal.pone.0219477

14. Bodini M, Brozzi A, Giuliani M, Nohynek H, Vainio A, Kuusi M, et al. Genomic characterization of invasive meningococcal serogroup B isolates and estimation of 4CMenB vaccine coverage in Finland. mSphere (2020) 5:10.1128/msphere.00376–20. doi: 10.1128/msphere.00376-20

15. Vogel U, Taha MK, Vazquez JA, Findlow J, Claus H, Stefanelli P, et al. Predicted strain coverage of a meningococcal multicomponent vaccine (4CMenB) in Europe: a qualitative and quantitative assessment. Lancet Infect Dis (2013) 13:416–25. doi: 10.1016/s1473-3099(13)70006-9

16. Hong E, Terrade A, Muzzi A, De Paola R, Boccadifuoco G, La Gaetana R, et al. Evolution of strain coverage by the multicomponent meningococcal serogroup B vaccine (4CMenB) in France. Hum Vaccin Immunother (2021) 17:5614–22. doi: 10.1080/21645515.2021.2004055

17. Clark SA, Willerton L, Claus H, Carannante A, Stefanelli P, Abad R, et al. Exploring the sequence diversity and surface expression of factor H-binding protein among invasive serogroup B meningococcal strains from selected European countries. Hum Vaccin Immunother (2024) 20:2427471. doi: 10.1080/21645515.2024.2427471

18. Tzanakaki G, Hong E, Kesanopoulos K, Xirogianni A, Bambini S, Orlandi L, et al. Diversity of Greek meningococcal serogroup B isolates and estimated coverage of the 4CMenB meningococcal vaccine. BMC Microbiol (2014) 14:111. doi: 10.1186/1471-2180-14-111

19. Tzanakaki G, Xirogianni A, Tsitsika A, Clark SA, Kesanopoulos K, Bratcher HB, et al. Estimated strain coverage of serogroup B meningococcal vaccines: a retrospective study for disease and carrier strains in Greece (2010–2017). Vaccine (2021) 39:1621–30. doi: https://doi.org/10.1016/j.vaccine.2021.01.073

20. Mulhall Robert M, Bennett D, Cunney R, Borrow R, Lucidarme J, Findlow J, et al. Potential coverage of the 4CMenB vaccine against invasive serogroup B *Neisseria meningitidis* isolated from 2009 to 2013 in the Republic of Ireland. mSphere (2018) 3:10.1128/msphere.00196–18. doi: 10.1128/msphere.00196-18

21. Carannante A, Fazio C, Neri A, Lista F, Fillo S, Ciammaruconi A, et al. Meningococcal B vaccine antigen FHbp variants among disease-causing *Neisseria meningitidis* B isolates, Italy, 2014–2017. PLoS One (2020) 15:e0241793. doi: 10.1371/journal.pone.0241793

22. Sereikaitė E, Plepytė R, Petrutienė A, Stravinskienė D, Kučinskaitė-Kodzė I, Gėgžna V, et al. Molecular characterization of invasive *Neisseria meningitidis* isolates collected in Lithuania (2009-2019) and estimation of serogroup B meningococcal vaccine 4CMenB and MenB-Fhbp coverage. Front Cell Infect Microbiol (2023) 13. doi: 10.3389/fcimb.2023.1136211

23. Bambini S, Piet J, Muzzi A, Keijzers W, Comandi S, De Tora L, et al. An analysis of the sequence variability of meningococcal fHbp, NadA and NHBA over a 50-year period in the Netherlands. PLoS One (2013) 8:e65043. doi: 10.1371/journal.pone.0065043

24. Freudenburg-de Graaf W, Knol MJ, van der Ende A. Predicted coverage by 4CMenB vaccine against invasive meningococcal disease cases in the Netherlands. Vaccine (2020) 38:7850–7. doi: https://doi.org/10.1016/j.vaccine.2020.10.008

25. Holst J, Comanducci M, Bambini S, Muzzi A, Comandi S, Oksnes J, et al. Variability of genes encoding surface proteins used as vaccine antigens in meningococcal endemic and epidemic strain panels from Norway. Vaccine (2014) 32:2722–31. doi: https://doi.org/10.1016/j.vaccine.2014.02.068

26. Waśko I, Gołębiewska A, Kiedrowska M, Ronkiewicz P, Wróbel-Pawelczyk I, Kuch A, et al. Genetic variability of Polish serogroup B meningococci (2010–2016) including the 4CMenB vaccine component genes. Vaccine (2020) 38:1943–52. doi: https://doi.org/10.1016/j.vaccine.2020.01.021

27. Bettencourt C, Nogueira P, Paulo Gomes J, João Simões M. Vaccines against *Neisseria meningitidis* serogroup B strains – What does genomics reveal on the Portuguese strain's coverage. Vaccine (2022) 40:4772–9. doi: https://doi.org/10.1016/j.vaccine.2022.06.038

28. Abad R, Medina V, Stella M, Boccadifuoco G, Comanducci M, Bambini S, et al. Predicted strain coverage of a new meningococcal multicomponent vaccine (4CMenB) in Spain: analysis of the differences with other European countries. PLoS One (2016) 11:e0150721. doi: 10.1371/journal.pone.0150721

29. Abad R, García-Amil C, Navarro C, Martín E, Martín-Díaz A, Vázquez JA. Molecular characterization of invasive serogroup B *Neisseria meningitidis* isolates from Spain during 2015-2018: evolution of the vaccine antigen factor H binding protein (FHbp). J Infect (2021) 82:37–44. doi: 10.1016/j.jinf.2021.01.030

30. Roca-Grande J, Mir-Cros A, Muñoz-Almagro C, Lung M, Bellés-Bellés A, Càmara J, et al. Genomic characterization of invasive *Neisseria meningitidis* in Spain (2011/12–2022/23): expansion of clonal complex 213 and the potential threat to 4CMenB vaccine strain coverage. Emerg Microbes Infect (2025) 14:2482696. doi: 10.1080/22221751.2025.2482696

31. Leo S, Lazarevic V, Girard M, Getaz-Jimenez Velasco GC, Gaïa N, Renzi G, et al. Strain coverage of Bexsero vaccine assessed by whole-genome sequencing over a cohort of invasive meningococci of serogroups B and W isolated in Switzerland. Vaccine (2020) 38:5324–31. doi: https://doi.org/10.1016/j.vaccine.2020.05.071

32. Parikh SR, Newbold L, Slater S, Stella M, Moschioni M, Lucidarme J, et al. Meningococcal serogroup B strain coverage of the multicomponent 4CMenB vaccine with corresponding regional distribution and clinical characteristics in England, Wales, and Northern Ireland, 2007-08 and 2014-15: a qualitative and quantitative assessment. Lancet Infect Dis (2017) 17:754–62. doi: 10.1016/s1473-3099(17)30170-6

33. Lucidarme J, Bai X, Lekshmi A, Clark SA, Willerton L, Ribeiro S, et al. Invasive serogroup B meningococci in England following three years of 4CMenB vaccination - first real-world data. J Infect (2022) 84:136–44. doi: 10.1016/j.jinf.2021.11.015

34. Rodrigues CMC, MacDonald L, Ure R, Smith A, Cameron JC, Maiden MCJ. Exploiting real-time genomic surveillance data to assess 4CMenB meningococcal vaccine performance in Scotland, 2015 to 2022. mBio (2023) 14:e0049923. doi: 10.1128/mbio.00499-23

35. Bettinger JA, Liberator P, Halperin SA, Vaudry W, Sadarangani M, Hao L, et al. Estimated susceptibility of Canadian meningococcal B isolates to a meningococcal serogroup B vaccine (MenB-FHbp). Vaccine (2020) 38:2026–33. doi: https://doi.org/10.1016/j.vaccine.2019.12.051

36. Meesters K, Sadarangani M, Clark SA, Borrow R, Tsang RSW, Le Saux N, et al. Characterization of Canadian *Neisseria meningitidis* serogroup B isolates and factor-H binding protein expression, data from the Canadian Immunization Monitoring Program Active (IMPACT), 2013-2020. Vaccine (2025) 55:127030. doi: 10.1016/j.vaccine.2025.127030

37. Rajam G, Stella M, Kim E, Paulos S, Boccadifuoco G, Serino L, et al. Meningococcal antigen typing system (MATS)-based *Neisseria meningitidis* serogroup B coverage prediction for the MenB-4C vaccine in the United States. mSphere (2017) 2:10.1128/msphere.00261–17. doi: 10.1128/msphere.00261-17

38. Chang H-Y, Vuong J, Hu F, Liberator P, Chen A, Kretz CB, et al. Distribution of *Neisseria meningitidis* serogroup b (NmB) vaccine antigens in meningococcal disease causing isolates in the United States during 2009-2014, prior to NmB vaccine licensure. J Infect (2019) 79:426–34. doi: 10.1016/j.jinf.2019.09.001

39. Muzzi A, Bodini M, Topaz N, Masignani V, Vadivelu K, Marjuki H, et al. Genetic features of a representative panel of 110 meningococcal B isolates to assess the efficacy of meningococcal B vaccines. mSphere (2022) 7:e00385–22. doi: 10.1128/msphere.00385-22

40. Efron A, Brozzi A, Biolchi A, Bodini M, Giuliani M, Guidotti S, et al. Genetic characterization and estimated 4CMenB vaccine strain coverage of 284 *Neisseria meningitidis* isolates causing invasive meningococcal disease in Argentina in 2010–2014. Hum Vaccin Immunother (2024) 20:2378537. doi: 10.1080/21645515.2024.2378537

41. de Filippis I, de Lemos APS, Hostetler JB, Wollenberg K, Sacchi CT, Harrison LH, et al. Molecular epidemiology of *Neisseria meningitidis* serogroup B in Brazil. PLoS One (2012) 7:e33016. doi: 10.1371/journal.pone.0033016

42. de Lemos APS, Sacchi CT, Gonçalves CR, Camargo CH, Andrade AL. Genomic surveillance of *Neisseria meningitidis* serogroup B invasive strains: diversity of vaccine antigen types, Brazil, 2016-2018. PLoS One (2020) 15:e0243375. doi: 10.1371/journal.pone.0243375

43. Arteta-Acosta C, Villena R, Hormazabal JC, Fernández J, Santolaya ME. Whole-genome sequencing of *Neisseria meningitidis* collected in Chile from pediatric patients during 2016–2019 and coverage vaccine prediction. Vaccine (2024) 42:126311. doi: https://doi.org/10.1016/j.vaccine.2024.126311

44. Alarcon ZK, Prada D, Gabastou JM, Sanabria O, Duarte C, Moreno J. Population structure of *Neisseria meningitidis* ST-9493 identified in Colombian isolates. Enfermedades infecciosas y microbiologia clinica (English ed) (2023) 41:290–3. doi: https://doi.org/10.1016/j.eimce.2022.01.005

45. PubMLST. Sequence attribute search - fHbp_peptide. https://pubmlst.org/bigsdb?db=pubmlst_neisseria_seqdef&page=alleleQuery&locus=fHbp_peptide&submit=1. Accessed January 2026.
